# Alternative TFAP2A isoforms have distinct activities in breast cancer

**DOI:** 10.1186/bcr2838

**Published:** 2011-03-04

**Authors:** Chiara Berlato, KaYi V Chan, Anna M Price, Monica Canosa, Angelo G Scibetta, Helen C Hurst

**Affiliations:** 1 Centre for Tumour Biology, Bart's Cancer Institute, Queen Mary University of London, Charterhouse Square, London EC1M 6BQ, UK

## Abstract

**Introduction:**

AP-2α is a transcription factor implicated in the regulation of differentiation and proliferation in certain tissues, including the mammary gland. In breast tumours, continued expression of AP-2α has been correlated with a better prognosis, but this is hard to reconcile with a reported role in the upregulation of the *ERBB2 *oncogene. The existence of *TFAP2A *isoforms, deriving from alternative first exons and differing in their N-terminal sequence, has been described in some mammals, but their relative abundance and activity has not been investigated in the human breast.

**Methods:**

Expression levels of four *TFAP2A *isoforms were assayed at the level of RNA and protein (via the generation of isoform-specific antibodies) in a panel of breast tumour cell lines and in tissue from normal breast and primary tumour samples. Expression constructs for each isoform were used in reporter assays with synthetic and natural promoters (cyclin D3 and *ERBB2) *to compare the activation and repression activity of the isoforms.

**Results:**

We demonstrate that the two isoforms AP-2α 1b and AP-2α 1c, in addition to the originally cloned, AP-2α 1a, are conserved throughout evolution in vertebrates. Moreover, we show that isoform 1c in particular is expressed at levels at least on a par with the 1a isoform in breast epithelial lines and tissues and may be more highly expressed in tamoxifen resistant tumours. The isoforms share a similar transactivation mechanism involving the recruitment of the adaptors CITED2 or 4 and the transactivators p300 or CBP. However, isoform 1b and 1c are stronger transactivators of the *ERBB2 *promoter than isoform 1a. In contrast, AP-2α 1a is the only isoform able to act as a repressor, an activity that requires an intact sumoylation motif present within the N-terminus of the protein, and which the other two isoforms lack.

**Conclusions:**

Our findings suggest that TFAP2A isoforms may be differentially regulated during breast tumourigenesis and this, coupled with differences in their transcriptional activity, may impact on tumour responses to tamoxifen therapy. These data also have implications for the interpretation of tumour studies that seek to correlate outcomes with TFAP2A expression level.

## Introduction

AP-2α belongs to the AP-2 family of transcription factors with four other members, AP-2β, γ, δ and ε [1], which have all been implicated in the regulation of proliferation and differentiation in specific tissues. In particular, AP-2α is expressed in the developing and adult mammary gland [2,3]. In breast cancer, lower AP-2α expression levels are found in invasive cancer compared to ductal carcinoma *in situ *(DCIS) and normal breast [4], while high levels of AP-2α correlate with a more favourable outcome [2,5]. Among the known target genes, many play a key role in breast biology and tumorigenesis. AP-2α is a central player in the positive regulation of *ERBB2 *expression [6], supported by studies demonstrating a correlation between AP-2α levels and expression of the receptor in tumour samples [5,7], but in conflict with other observations [4]. How the role of AP-2α as a tumour suppressor reconciles with its activity in inducing *ERBB2 *is still unclear.

AP-2 proteins interact as homo- and hetero-dimers which bind to specific GC-rich sequences to regulate transactivation or repression [8]. The best characterised mechanism of transactivation involves the recruitment of the transcriptional activators CBP and p300 through interaction with the small adaptor proteins CITED2 [9] or CITED4 [10]. The importance of these interactions *in vivo *is underlined by the observation that CITED2 and AP-2α knockout mice have overlapping phenotypes [11]. AP-2α is known to repress expression of a number of genes, including C/EBPα [12], Bcl-2 [13], EGFR [14], but the mechanism of repression is unknown. However, the related AP-2γ is known to interact with UBC9 and to be sumoylated, resulting in downregulation of its transcriptional activity [15].

The *TFAP2A *gene consists of seven exons with the last six exons encoding the majority of the protein, including the activation, DNA binding and dimerisation domains [1]. The existence of different TFAP2A isoforms deriving from alternative first exons has been described in murine embryo and HeLa cells [16], and in ovine and human placenta [17]. Some variation in spatio-temporal expression between the isoforms during murine embryonic development was identified using *in situ *hybridisation [16]; however, the function of these splice variants, which differ solely in the extreme amino-terminal sequence of AP-2α, has not been investigated further. Generally, transcripts deriving from alternative first exons are frequently observed in mammalian genomes, with an estimated >20% of genes having active alternative promoters [18]. This contributes to the complexity of the function of a gene by providing additional levels of regulation of expression and, if the translation start site exists within the first exon as in the case of AP-2α, by encoding distinct proteins which potentially have quite different biological functions.

Our aim was to characterise the role of human AP-2α isoforms in normal breast epithelial cells and tumours, addressing their expression levels and comparing their transcriptional activities.

## Materials and methods

### Additional sequences used to analyse TFAP2A isoform conservation

*Xenopus tropicalis *(1a: NM_001037258.1, 1b: BC135698, 1c: CR848274.2); *Danio rerio *(1a: NM_176859.2, 1b: BC066582, 1c: AF457192.1).

### Antibodies and plasmids

AP-2α isoform-specific antibodies were generated utilising the peptides representing the different isoforms (1a: KLTDNIKYEDCEDRHDGTSN, 1b: LVHSFSAMDRHDC, 1c: SILAKMGDWQDRC 1d: GGARGQTGPGSAC), coupled to the KLH carrier protein, injected in rabbits according to standard protocols. The specificity of the antibodies was tested in Western blot on extracts of HepG2 overexpressing the different isoforms. Isoform 4 serum was specific and was further used in a dilution 1:100. Other isoform-specific antibodies were further purified utilising the peptides KLTDNIKYEDCEDRC (1a), LVHSFSAMDRC (1b), and SILAKMGDWQDRC (1c) coupled to a SulfoLink Coupling Gel Column (Thermo Fisher Scientific Inc, Rockford, IL, USA)), according to the manufacturer's instructions, eluted with the corresponding peptides, and concentrated. The isoform 1a antibody was further incubated on a SulfoLink column conjugated to the peptide (DRHDGTSNGTARLC) to retain exon 2 binding antibodies. Pan-AP-2α (3B5), actin and HSC70 antibodies were from Santa Cruz Biotechnology Inc. (Santa Cruz, CA, USA). pcDNA3 encoding full length human AP-2α isoform 1a [19] was mutagenised to the WT sequence (V2L) using the GeneEditor kit (Promega UK, Southampton, UK). Expression plasmids for isoforms 1b, 1c and 1d were generated in the same background. The N-terminal portion of the cDNA encoding AP-2α isoform 1c was generated by PCR with the primers GATGTCCATACTTGCCAAAATGG and TGAGGTACATTTTGTCCATGGC from cDNA generated from MCF10A cells and sublcloned by KpnI-BlpI digestion into pcDNA3 AP-2α, thus substituting the 5' sequence of isoform 1a. pcDNA3 AP-2α isoform 1b was generated by substituting the HindIII-BlpI fragment of pcDNA3-AP-2α-1a with the EarI-BlpI fragment of IMAGE clone 4432023. pcDNA3 AP-2α isoform 1d was generated by substituting the fragment HindIII-BlpI of pcDNA3-AP-2α-1a with the SfoI-BlpI fragment from IMAGE clone OE37H07. All constructs were verified by sequencing. Isoform 1a was further mutagenised (K10R) using the QuickChange kit (Agilent Technologies UK Ltd, Wokingham, UK). Other plasmids were: pCI-p300 [20], pRc/CMV CBP [21], pcDNA3-CITED2 [11], pcDNA3-CITED4 [10], pSG5 sumo1 ΔHSTV and pSG5 sumo2 ΔVY generated by PCR, pSG5 ubc9 [15], pSG5 Ubc9 C93S [15]. Reporter plasmids were: 3xAP2-Bluc [11], p500LUC, generated by subcloning the -500/+40 *ERBB2 *promoter fragment from p500CAT [22] into pGL3-basic (Promega), and cyclin D3 generated by cloning the region (-201,+162) into pGL3 basic. For normalisation, reporters expressing Renilla luciferase (pGL4.74 hRluc/TK or phRG-TK; Promega) were utilised.

### Cell culture, transfection and cell extracts

All cell lines were from the ATCC and were cultivated according to the criteria provided: HepG2 cells - DMEM supplemented with 10% foetal calf serum, penicillin and streptomycin; MCF10A - DMEM:F12 1:1 supplemented with EGF, hydrocortisone, cholera toxin, insulin, 5% horse serum, penicillin and streptomycin [23]. Tamoxifen-resistant cell lines were cultivated in 10^-7 ^M tamoxifen for six months. Cells were transiently transfected with GeneJuice (Merck KGaA, Darmstadt, Germany) in a 24-well format for luciferase experiments (80,000 cells/well) or 6-well format for Western blot experiments (350,000 cells/well) using a 1:3 ratio DNA:Genejuice. The total amount of DNA transfected was normalised using the appropriate empty vectors. Cells were harvested after 48 h, lysed and assayed using the dual luciferase reporter system (Promega). Firefly luciferase readings were normalised to renilla luciferase values. All transfection experiments were performed in triplicate and repeated at least three times; the average, with the SE, is shown. Statistical analysis was performed utilising Student's *t*-test. Extracts for Western blot were generated utilising RIPA buffer (50 mM Tris-HCl pH 7.5, 150 mM NaCl, 0.5% sodium deoxycholate, 0.1% SDS, 1% Nonidet P-40). Where indicated, isopeptidase inhibitors were included at 10 mM iodoacetamide (IAA) and 20 mM N-ethylmaleimide (NEM). Alternatively, cell pellets were resuspended directly in urea buffer (8 M urea, 1 M thiourea, 0.5% CHAPS, 50 mM DTT, 24 mM spermine). Western blot quantifications were achieved using ImageJ software [24], using a *t*-test for statistical analysis.

### RNA preparation and RT-PCR

RNA was extracted using the RNAeasy kit (Qiagen UK, Crawley, UK). We used 1 μg of total RNA to reverse transcribe with Superscript III (Life Technologies Corporation, Carlsbad, CA, USA). Real-time PCR of the different isoforms was performed using SybrGreen (Life Technologies) according to standard cycling protocols with 300 nM of specific forward primer (isoform 1a: ATATCAAGTACGAGGACTGCG, isoform 1b: AGATGTTAGTTCACAGTTTTTCAGC, isoform 1c: GATGTCCATACTTGCCAAAATGG, isoform 1d: CCAAGCAGCTCCTACC), and 50 nM of common reverse primer TTGCGACTGGGGGTAGATAG. Absolute amounts of each isoform were determined by generating a standard curve from a known amount of each linearised pcDNA3-AP-2α plasmid.

### Tumour samples

Fresh frozen tissue from 11 ER + ve patients was obtained from Guys and St Thomas/King's College London (GSTFT/KCL) Breast Tissue Bank (Ethics approval number LREC Ref 06/Q0603/25). All were treated with tamoxifen after surgery; tamoxifen resistant patients had relapsed within two years. Normal breast tissue samples were provided by Prof. Louise Jones (LREC Ref 05Q403/199). Statistical analysis was performed using the Mann-Whitney test.

## Results

### *TFAP2A *alternative first exons are conserved in vertebrates

**Figure 1:**
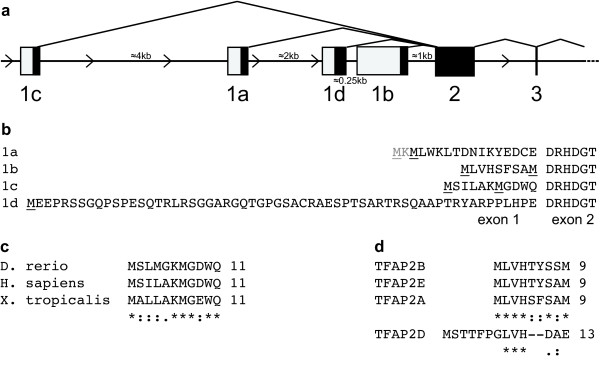
***TFAP2A *gene contains four alternative first exons which encode conserved protein sequences**. **(a) **Schematic representation of TFAP2A 5' gene structure (exons 1 to 3). Exons are shown as rectangles, introns as horizontal lines, drawn in proportion to their actual length. Closed rectangles represent translated regions, open rectangles represent untranslated regions. Isoforms 1b and 1c are orthologous to the murine isoform 3 [16] and ovine variant 6 [17], respectively. **(b) **TFAP2A protein sequences encoded by the four alternative first exons. Possible starting methionine residues are underlined. AP-2α isoform/variant 4 (as described in [16] and [17], respectively), generated by initiation of transcription upstream of exon 2, does not have a human paralog due to the presence of an in-frame stop codon upstream of exon 2. **(c) **Alignment of TFAP2A isoform 1c protein sequences in *H. sapiens*, *D. rerio *and *X. tropicalis *generated by ClustalW. Sequences for rhesus, mouse, dog and elephant are identical to the human sequence. * indicates an identical amino acid; : and . indicate conserved and semi-conserved substitutions, respectively. A conserved TATA-box is present 237 bp upsteam of the ATG. **(d) **Alignment of TFAP2A isoform 1b protein sequence to TFAP2B isoform 1b (EST: BM727695), TFAP2D (NM_172238.3) and TFAP2E (NM_178548.3) generated by ClustalW. Human sequences are shown.

The human *TFAP2A *gene structure and expressed sequence tags (ESTs) were analysed and the existence of three isoforms, assigned as reference sequences named isoform 1a, 1b, and 1c, was confirmed (Figure 1a, b). In addition, the existence of a fourth isoform, whose exon is located between exons 1a and 1b was suggested (Figure 1a). AP-2α isoform 1a corresponds to the AP-2α cDNA originally cloned [19] and is represented by nine ESTs. AP-2α isoforms 1b and 1c are represented respectively by 10 and 8 ESTs. The fourth splice variant, which we named isoform 1d, is represented by three ESTs, only one of which, derived from a corneal cDNA library, is correctly spliced.

Alternative transcripts are not always functional, but a criterion that suggests biological activity is conservation across species. Our analysis revealed that highly conserved homologs of AP-2α 1a and 1b are found in mammals and in *Xenopus tropicalis *and *Danio rerio*. AP-2α 1c is also well conserved in mammals, and has a homolog in *X. tropicalis *and *D. rerio*, although with a lower sequence conservation (Figure 1c). This conservation of the isoforms across species suggests they have been under positive selective pressure and hence have distinct roles required for normal development. The one exception may be 1d since the sequence and structure of this exon is conserved only in primates; therefore, given that this isoform is represented by only one correctly spliced EST, its existence is more questionable.

All the alternative first exons identified encode for at least one possible initiator methionine. Exon 1a, 1b and 1c each encode for a very short sequence of amino acids, resulting in proteins with a very similar predicted molecular weight, while exon 1d encodes for a longer N-terminus (60 residues) (Figure 1b). All the reported isoforms - with the exception of 1d - have two in-frame alternative methionine residues (Figure 1b). For isoform 1a, the starting methionine was determined to be the downstream one by amino-terminal peptide sequencing when the protein was originally purified [19]. For isoforms 1b and 1c, since both methionine residues are conserved across different species, we searched for a Kozak sequence to help predict the relative strength of each translation initiation site [25]. This suggested that for isoform 1b, the downstream ATG may encode the predominant starting methionine residue. However, we could not differentiate between the two ATGs in isoform 1c since both have a conserved purine at position -3.

### TFAP2A isoforms 1a and 1c are expressed at a similar level in breast cell lines and tissue

**Figure 2:**
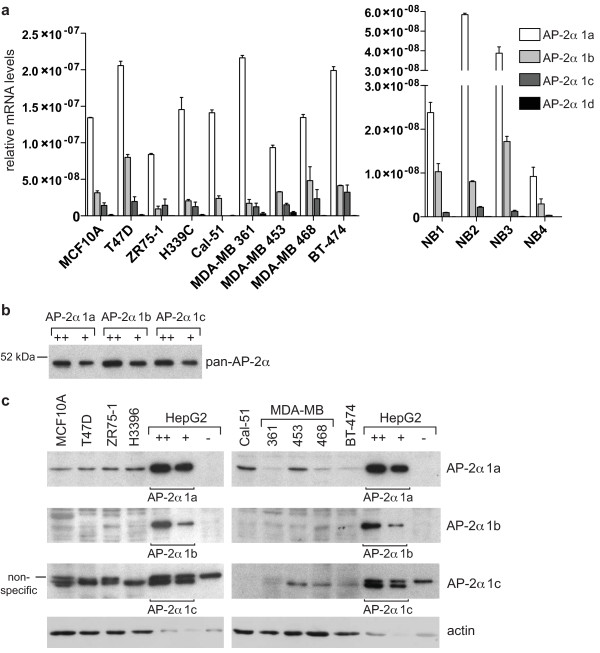
**AP-2α isoforms 1a, 1b, and 1c are expressed in breast cell lines and tissues**. **(a) **cDNA was generated from the indicated breast cell lines (left) and normal breast samples (right) and the relative amount of the different AP-2α isoforms was determined by real-time PCR as described in the Materials and methods. **(**b**)** HepG2 cells were transfected with pcDNA3 expression vectors encoding each isoform and harvested after 48 h. Two different amounts of cell lysate (5 and 2.5 μg) were loaded for each isoform. Western blot analysis was performed with a pan-AP-2α antibody, 3B5, which recognises an epitope in the DNA-binding domain of the protein common to all isoforms. **(c) **Protein lysates (30 μg) derived from the indicated cell lines were analysed by western blot with isoform-specific antibodies. As a loading control, two amounts of lysate from HepG2 cells overexpressing each isoform from (b) were loaded on each gel and the blots were also reprobed for actin (lower panels).

In order to examine the relative expression of the different isoforms in breast tissue, a specific real time PCR assay was set up to discriminate between each of the amino-terminal variants. This analysis revealed that isoform 1a is the predominant isoform at the RNA level in a panel of different breast cell lines and four samples of normal breast tissue (Figure 2a). A significant, albeit lower, expression of isoforms 1b and 1c was also detected in the cell lines and the tissue samples (Figure 2a). In contrast, isoform 1d levels were very low, suggesting that this isoform, if it exists, does not play a prominent role in the breast. 1d expression was investigated additionally in an RNA sample from human foetal eye where, again, the mRNA levels were undetectable (not shown).

To investigate expression at the protein level, isoform specific antibodies were generated (as described in Materials and methods). An antibody affinity analysis was performed by transfecting HepG2 cells, which do not express detectable levels of AP-2α, with specific expression plasmids generated for each isoform, under the control of the CMV promoter. The expression levels of the different isoforms, as assessed using an antibody against the common DNA binding domain of the protein (pan-AP-2α, 3B5), were consistently similar (Figure 2b). The same quantity of each lysate was used as a loading control for subsequent isoform-specific Western blots (Figure 2b). AP-2α 1a and 1c antibodies bound to their respective isoform with high affinity, while the 1b-specific antibody showed significantly lower affinity (compare Figures 2b and 2c). Western blot analysis of a panel of breast tumour lines showed that isoform 1a was expressed at modest levels in all the cell lines investigated (Figure 2c). A faint band corresponding to the molecular weight of isoform 1b could be detected in all the cell lines, but the low affinity of the antibody made it difficult to reliably distinguish it above the background (Figure 2c). In contrast, isoform 1c was expressed at significant levels in all the cell lines investigated, with the exception of Cal51, which also lacked detectable expression at the mRNA level (Figure 2a, c). Isoform 1d protein was not detected in any of the cell lines (not shown), in accordance with the mRNA data. Comparing the intensity of signal between the breast line and transfected HepG2 control lysates, suggested that protein levels of isoform 1c are at least comparable to those of isoform 1a in many lines. This was examined graphically, by scanning the Western blots (see Figure S1 in Additional File 1), which demonstrated that isoform 1c is expressed at significantly higher levels in the majority of lines examined, including MCF10A, T47D, ZR75-1, MDA-MB 361 and MDA-MB 468 cells. Therefore, the protein levels of isoforms 1a and 1c are much more similar than would be inferred from the mRNA data alone and this disproportion between mRNA and protein levels suggests that these two isoforms are differentially regulated either at the translational or post-translational level.

### TFAP2A isoforms share a similar transactivation mechanism

We next explored whether the isoforms have distinct biological properties. The DNA binding activity of the isoforms was compared using electromobility shift assays (EMSA) and nuclear extracts from HepG2 cells transfected for each of the different isoforms. This revealed that they all have a similar affinity for a consensus AP-2 DNA binding site (Figure S2 in Additional File 1). All the isoforms showed a high stability, without detectable differences in half-life when exogenously expressed in HepG2 cells (not shown).

**Figure 3:**
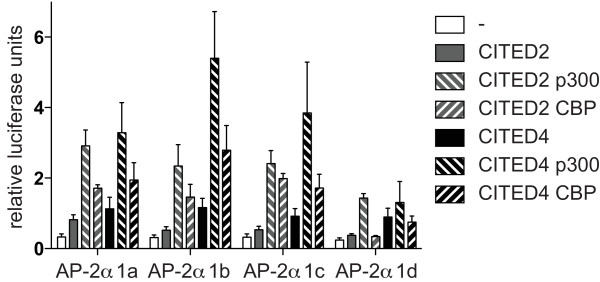
**AP-2α isoforms exert similar transactivation activity when cotransfected with CITED2 or 4 and p300 or CBP**. HepG2 cells were transfected with 0.05 μg pcDNA3-AP-2α, 0.25 μg 3xAP2-Bluc, 0.25 μg phRG-renilla, 0.25 μg pcDNA3-CITED2/4, 1 μg pCI-p300 or pRc/CMV CBP as indicated. Relative firefly luciferase activity normalised to renilla luciferase activity is shown. Average and standard error from four independent experiments is reported.

To determine whether all the isoforms can act as transactivators in combination with CITED2 or 4 and CBP or p300, their activity was analysed in HepG2 cells cotransfected with a synthetic AP-2 dependent luciferase reporter construct widely used to characterise AP-2 activity [11]. Most expression constructs for the 1a isoform carry a mutation at the second amino acid (V2L), introduced when AP-2α was first cloned [19], which we mutated back to the wild type sequence in order to accurately compare the isoforms. All showed similar basal levels of transactivation activity (Figure 3, open boxes). When cotransfected with the adaptors CITED2 or CITED4, all isoforms gave enhanced activation of the reporter (*P *< 0.05) compared to the control (Figure 3, grey and black boxes). Cotransfection with p300 or CBP in combination with the different CITED factors resulted in a further increase in transactivation activity (*P *< 0.05) for isoforms 1a, 1b, and 1c (Figure 3). An exception was isoform 1d, which did not further transactivate the reporter in the presence of CBP. Moreover, transactivation levels for this isoform were lower compared with the others. Taken together, these data suggest that all isoforms (excluding 1d) are able to interact functionally with the adaptors CITED2 or CITED4 with similar efficiency leading to the recruitment of p300 or CBP.

### TFAP2A 1a represses the cyclin D3 promoter via a sumo-dependent mechanism

**Figure 4:**
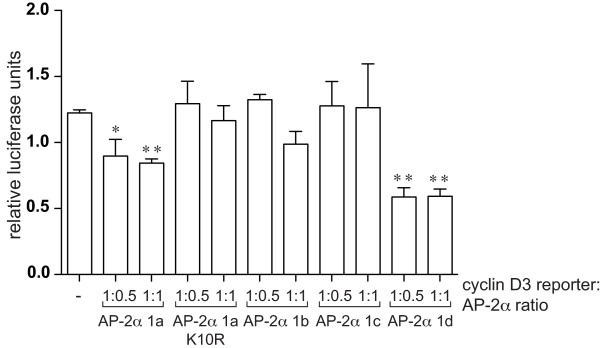
**AP-2α isoforms differ in their transcriptional repression activity**. HepG2 cells were transfected with 0.25 μg/well pGL4.74 (Renilla), 0.4 μg/well cyclin D3 reporter construct, and the indicated ratios of pcDNA3-AP-2α (corresponding to 0.2 and 0.4 μg/well). Results are reported as relative firefly luciferase activity normalised to renilla luciferase activity. Average and standard error from three independent experiments is shown.

Transcriptional activity of the isoforms was further compared using natural promoters from genes known to be regulated by AP-2α, including those shown to be repressed by AP-2 factors. We have observed that AP-2α downregulates cyclin D3 expression, through direct binding to sites within the proximal promoter (KV Chan and HC Hurst, unpublished). The activity of AP-2α isoforms on the cyclin D3 promoter was compared in HepG2 cells transfected with a reporter comprising the minimal promoter region (-201/+162) able to drive cyclin D3 expression. Isoforms 1b and 1c failed to exert any effect on the cyclin D3 promoter at any ratio of reporter: expression construct tested (Figure 4). In contrast, isoform 1a had a reproducible and significant repressive effect (Figure 4). Isoform 1d also exerted a very pronounced inhibitory effect, up to 50% (Figure 4). The inhibitory effect at a 1:1 ratio was already maximal for isoforms 1a and 1d, and did not change at higher ratios for isoforms 1b or 1c (not shown).

**Figure 5:**
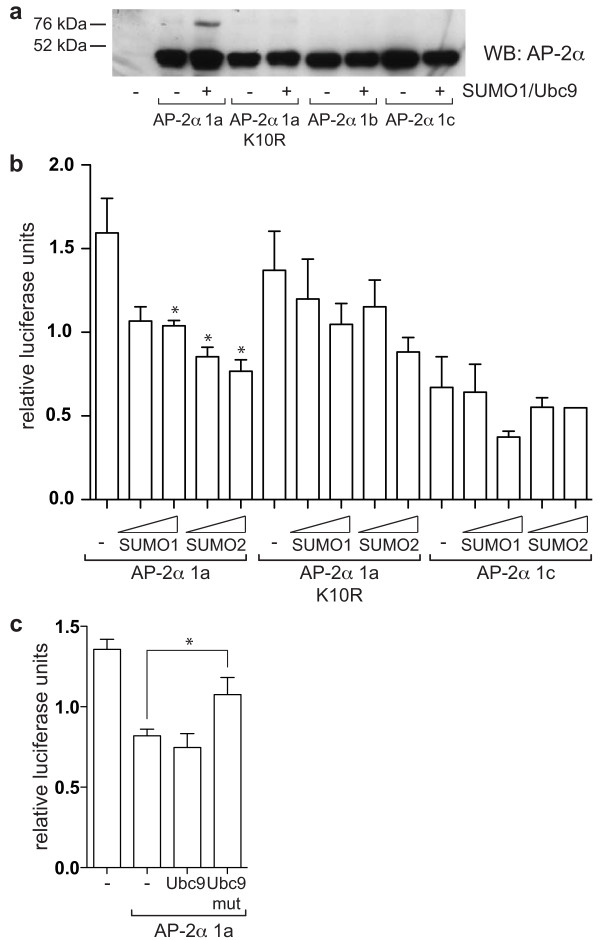
**AP-2α isoform 1a can be sumoylated leading to decreased transactivation activity**. **(a) **HepG2 cells were transfected with 0.3 μg/well of the different pcDNA3-AP-2α constructs, without and with 0.1 μg/well pSG5 Ubc9 and 0.6 μg/well pSG5 SUMO1 in a six-well format. 48 h after transfection, lysates were harvested in RIPA buffer containing IAA and NEM, and analysed by western blot with a pan AP-2α antibody (3B5). One experiment representative of three is shown. A second, weaker sumoylation site (IKKG) was predicted (using "SUMOplot") within the C-terminal half of the protein which could explain the low levels of sumoylation observed for AP-2α K10R and the other isoforms using longer exposures. **(b) **HepG2 cells were transfected with 0.05 μg/well of the different pcDNA3-AP-2α constructs, 0.25 μg/well 3xAP2-Bluc, 0.25 μg/well phRG-renilla, 0.25 μg/well pcDNA3-CITED2, 0.75 μg/well pCI-p300, and 0.25 or 0.5 μg/well pSG-SUMO1/2 as indicated. Relative firefly luciferase activity normalised to renilla luciferase activity is shown. The average and standard error of three experiments is reported. **(c) **HepG2 cells were transfected with 0.2 μg/well pGL4.74 (Renilla), 0.3 μg/well cyclin D3 reporter, 0.15 μg/well pcDNA3-AP-2α isoform 1a, 0.3 μg/well pSG-Ubc9 and 0.15 μg/well pSG-SUMO1. Average and standard error from three independent experiments is represented.

A key difference between AP-2α isoform 1a and the other two isoforms expressed in breast is the presence of a putative sumoylation site (IKYE) centred at lysine 10 within the sequence encoded by exon 1a. This site is homologous to the site found in AP-2γ which has previously been demonstrated to be conjugated to SUMO *in vivo *thereby reducing AP-2γ transactivation activity [15]. Consequently, we hypothesised that this post-transcriptional modification may also play a role in the negative regulation of the cyclin D3 promoter by AP-2α isoform 1a. To test this, a non-sumolylalable mutant of isoform 1a, K10R, was generated and found to lack inhibitory activity on the cyclin D3 reporter construct (Figure 4). AP-2α has previously been demonstrated to interact with the SUMO E2-conjugating enzyme, UBC9, in a yeast two-hybrid screen [15], but to confirm that AP-2α can be sumoylated *in vivo*, expression constructs for UBC9 and SUMO-1 (Figure 5a) or SUMO-2 (not shown) were cotransfected in HepG2 cells together with the different AP-2α isoforms. This resulted in the appearance, for isoform 1a only, of a slower migrating band at approximately 75 kDa, compatible with a mono-sumoylated form of AP-2α (Figure 5a). Furthermore, mutation of lysine 10 led to a very pronounced reduction in intensity of this sumoylated band, thus confirming that lysine 10 is the predominant sumoylation site in isoform 1a, and explaining why the other isoforms were not significantly modified.

To confirm that the inhibitory activity of isoform 1a is dependent on sumoylation, HepG2 cells were cotransfected with the 3xAP2-Bluc reporter and increasing doses of SUMO-1 or SUMO-2. The transactivation activity was reduced with SUMO co-transfection, particularly by SUMO-2, while the transactivation activity of the K10R mutant or AP-2α 1c was not altered significantly, suggesting that the most important sumoylation site is indeed lysine 10 of isoform 1a (Figure 5b). To confirm that the inhibitory effect exerted by isoform 1a on the cyclin D3 reporter is due to sumoylation, we co-transfected with either wild-type UBC9 or its sumoylation defective mutant, C93S [26]. While transfection of wt UBC9 did not result in further inhibition of cyclin D3 reporter activity, cotransfection of UBC9-C93S partially reverted the inhibitory activity of isoform 1a (Figure 5c). In addition, a clear reduction in the level of sumoylated AP-2α was observed by western blot when the 1a isoform and UBC9-C93S were co-expressed in the same proportion in HepG2 cells (Figure S3 in Additional File 1).

### TFAP2A isoform 1a is a weaker transactivator of the *ERBB2 *promoter

**Figure 6:**
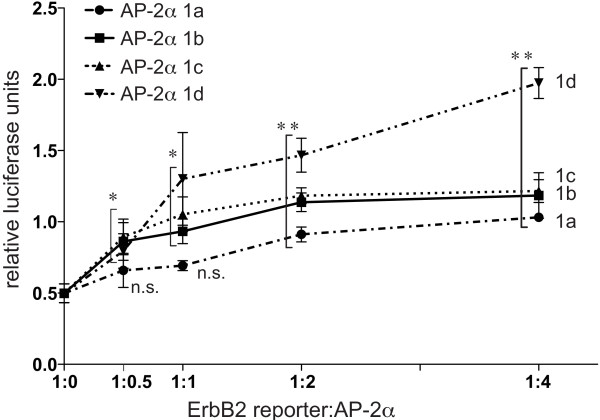
**AP-2α isoforms exert different levels of transactivation activity at the *ERBB2 *promoter**. HepG2 cells were transfected with 0.12 μg/well p500 ErbB2-luc, 0.02 μg/well renilla hRG, 0.06 μg/well pCI-p300, 0.06 μg/well pcDNA3-CITED2, and indicated ratios (corresponding to a total of 0.06, 0.12, 0.24 and 0.48 μg/well) of the different pcDNA3-AP-2α constructs. The average and standard error of three independent experiments is represented.

The transactivation activity of the different AP-2α isoforms was further tested on the *ERBB2 *promoter, which is known to possess two functional AP-2 binding sites, mapping at -210 and -500 upstream of the transcription start site, which are required for positive regulation of *ERBB2 *by AP-2α in breast tumour lines [22,27]. A reporter carrying *ERBB2 *promoter sequences (-500/+40) was co-transfected at different ratios with the AP-2α isoform expression constructs together with Cited2/p300 (Figure 6). Isoform 1a significantly induced reporter activity at a 1:2 and 1:4 reporter:AP-2α ratio (Figure 6). In contrast, the transactivation activity of isoforms 1b and 1c was already significant at a ratio of 1:0.5, and further increased at higher ratios, reaching a plateau at 1:4. Isoform 1d was the most potent transactivator and its activity increased at higher ratios in an almost linear manner. Consequently, it appears that isoform 1a is the weakest transcriptional activator on the *ERBB2 *promoter, Thus, when tested in the context of a complex, natural promoter, differences in transactivation activity between the TFAP2A isoforms are observed which were not evident when using the artificial reporter construct (Figure 3). To examine this further, the relative activity of isoforms 1a, 1b and 1c on the *ERBB2 *promoter was also compared in the presence of different combinations of the CITED/p300/CBP cofactors (Figure S4 in Additional File 1). In each case, isoform 1a was the weakest activator.

### TFAP2A isoform 1c expression increases in tamoxifen-resistant lines and tumours

**Figure 7:**
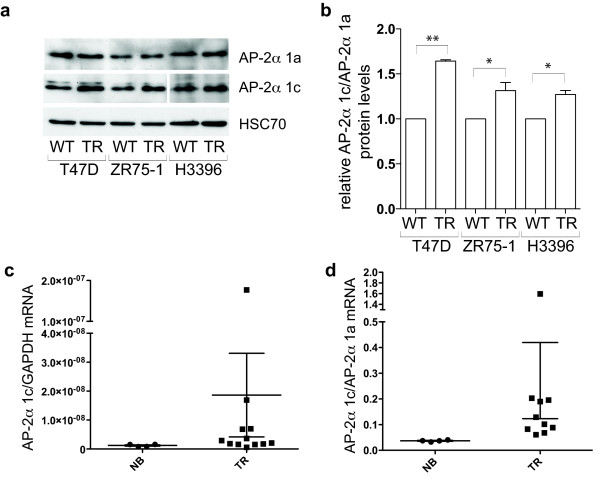
**AP-2α isoform expression is differentially regulated in tamoxifen resistant cell lines and breast tumour samples**. **(a, b)**: Levels of AP-2α isoform 1a and 1c were determined by Western blot in a series of wild-type ER+ breast tumour lines and in their tamoxifen-resistant counterparts. The signal from a number of different non-saturated exposures was quantified with ImageJ and is graphically represented in (b). **(c, d)**: Normal breast (4) and tumour samples (10) were analysed by real-time PCR for AP-2α isoform expression levels. The relative levels of AP-2α 1c normalised using GAPDH levels (c) and the ratio between AP-2α 1c and 1a (d) are represented. The differences remain statistically significant even when the outliers are eliminated from the analysis.

Since we observed that AP-2α isoforms have differential transactivation activity on the *ERBB2 *promoter, we decided to investigate their expression in a biological context. ErbB2 overexpression is associated with resistance to the oestrogen receptor (ER) antagonist tamoxifen, since signalling from the receptor can promote oestrogen independent activation of the ER (reviewed [28]). We, therefore, investigated whether AP-2α isoform levels were altered in tamoxifen resistant (TR) lines and tumour samples. Given that isoform 1a was the weakest activator of *ERBB2 *expression (Figure 6), we would predict that increased expression of the other isoforms might be associated with the tamoxifen resistant phenotype. In three independent TR lines, which were cultivated in the presence of tamoxifen for at least six months, we observed a pronounced increase in levels of ErbB2 compared to the wt controls (Figure S5a, b in Additional File 1). In the same three lines, levels of isoform 1c compared to isoform 1a increased significantly, as measured by quantifying levels in Western blots as the ratio between the two isoforms (1c:1a; *P *< 0.05, see Figure 7a, b).

This finding led us to compare *TFAP2A *isoform expression levels in a small series of mRNA samples from TR tumours. Good quality RNA from frozen samples was required to detect isoforms levels with sufficient sensitivity, and this limited the number of samples available. In these samples, *ERBB2 *mRNA levels tended to be higher compared to normal breast samples (Figure S3c in Additional File 1; *P *= 0.043). AP-2α isoform 1c levels were also higher in TR tumours compared to normal breast (Figure 7c, *P *= 0.038). No significant change was observed for isoform 1a or 1b. Moreover, the difference in ratio between levels of isoform 1c and 1a in the TR tumours compared with normal breast was also significant (Figure 7d, *P *= 0.002). This would suggest a selective and differential regulation of AP-2α isoforms in tamoxifen resistant tumours.

## Discussion

A significant proportion of the reports investigating the biological function of AP-2α are overexpression studies which analyse exclusively the first isoform (1a) cloned. The existence of additional TFAP2A transcripts deriving from alternative first exons has been described in some mammals, but there has been little attempt to determine their importance or function. Our *in silico *analysis confirmed that different isoforms of AP-2α occur in humans, providing at the same time evidence of a strong conservation throughout vertebrate evolution (Figure 1). Furthermore, ESTs for isoforms 1a and 1b can also be found for the paralogous gene AP-2β. In contrast, we were not able to identify alternative 5' variants for AP-2γ, which is the homolog of AP-2α isoform 1a. Interestingly, AP-2ε, and possibly the more distantly related AP-2δ, are more similar to isoform 1b (Figure 1d), suggesting this is the most ancient isoform from which the family originated in vertebrates. Since we were unable to detect expression of isoform 1d in the tissues examined, it probably does not represent a true isoform. However, the remaining AP-2α isoforms are expressed at significant levels in breast cell lines and tissue (Figure 2) with the AP-2α 1c protein being found at levels at least comparable to those of the initially identified isoform 1a, suggesting that its activity in the breast should be investigated.

Our analysis focused on testing whether differences in amino-terminal sequence lead to distinct biological characteristics for the AP-2α isoforms. The N-terminus of AP-2α encompasses the transactivation domain, and is not involved either in DNA binding or in protein dimerisation [1]. Therefore, functional differences are more likely to be found in transactivation activity, although the limited difference in sequence might suggest subtle variations. With a synthetic reporter, isoforms 1a, 1b and 1c showed similar transactivation activity indicating a similar ability to interact functionally with CITED2 or CITED4 and p300 or CBP. This agrees with the finding that the domain essential for interaction with CITED2 lies in the central (common) region of AP-2α [9]. However, the picture changed when the transactivation activity of the isoforms was compared using natural promoters. The cyclin D3 promoter was differentially regulated by the different isoforms with AP-2α 1b and 1c having a minimal effect, whereas isoform 1a exerted a significant inhibitory activity, similar to the effect exerted by AP-2γ (KV Chan and HC Hurst, unpublished). Lysine 10, which lies within a putative sumoylation motif, was essential for this inhibitory activity. An interaction between AP-2α and UBC9 has been demonstrated previously [15] and our subsequent experiments showed that isoform 1a alone could be sumoylated in HepG2 cells. However, sumoylation of endogenous AP-2α in breast lines could not be confirmed. When a similar experiment was performed for AP-2γ using MCF7 cells, which express it at high levels, only a small fraction of the protein was found to be sumoylated [15], in accord with similar studies on other sumoylated proteins [29]. By extrapolation, since in breast lines levels of isoform 1a represent only a proportion of the total AP-2α protein (Figure 2), it is likely that the fraction of sumoylated isoform 1a falls below the level detectable using Western blotting or immunoprecipitation. Co-transfection of SUMO-1 or SUMO-2 with isoform 1a resulted in reduced transactivation activity, similar to observations made for AP-2γ [15]. The finding that for AP-2α only isoform 1a can act as a repressor strengthens the hypothesis that sumoylation is necessary for AP-2 transcriptional repression. Moreover, we have linked for the first time the negative regulatory effect on the natural cyclin D3 promoter to sumoylation of AP-2α, since overexpression of mutant Ubc9 reverted the inhibitory activity of isoform 1a.

On the *ERBB2 *promoter, isoforms 1b and 1c both exerted significant transactivation activity at low reporter:expression plasmid ratios (Figure 6), while isoform 1a only achieved a similar level of transactivation activity when two to four times more expression plasmid was used, despite very similar expression efficiencies for all three expression constructs. This was initially explored using CITED2/p300 cofactors since CITED2 is considered to be the most biologically relevant cofactor for TFAP2A due to the similarity in phenotype between the respective knock-out mice. Moreover, a recent genome-wide analysis of CBP and p300 binding suggested that p300 is significantly more associated with AP-2 sites compared to CBP [30]. However, we have also compared cofactor preferences for the TFAP2A isoforms on both the synthetic and *ERBB2 *promoters at a variety of ratios (Figures 3, 6, S4 and data not shown) and noted that CITED4 also acts efficiently with isoforms 1b and 1c. However, with all cofactor combinations, isoform 1a was consistently the weakest transactivator at the natural *ERBB2 *promoter. It is significant, therefore, that we have observed that isoform 1c levels are higher in tamoxifen resistant breast tumour samples and cell lines. Although this observation has to be treated with some caution because of the limited number of samples available to assay, it suggests that AP-2α isoforms may have differential roles in tumourigenesis due to variations in their transactivation activity on key target genes such as *ERBB2*.

These data demonstrate that the short sequence of amino acids encoded by the first exons affects significantly the transactivation potential of AP-2α. In particular isoform 1a may be a weaker transactivator and, potentially, has a more specialised function as an inhibitor of transcription on AP-2α repressed genes. The lack of repressor activity shown by isoforms 1b and 1c may be explained by the absence of the sumoylation motif. Alternatively, the sequence encoded by these isoforms may mediate an enhanced activation function that masks any inhibitory activity. Isoform 1c possesses a well conserved lysine residue, which is a possible target for a variety of regulatory post-translational modifications, including ubiquitination, acetylation and methylation, which will be the object of further investigations.

## Conclusions

The different isoforms of AP-2α possess differential transactivation and repression activities which are dependent on the promoter context. AP-2α isoform 1c is expressed at significant levels in breast cell lines and tissues, and can be the predominant isoform. These observations underline the need to determine which isoforms are expressed at the protein level in different tissues and tumours, and that the complexity of the different AP-2α isoforms has to be considered when conducting promoter studies on AP-2 target genes. Moreover, further studies are needed to analyse AP-2α isoform 1c function in the breast and during tumourigenesis. Indeed, differences between the isoforms may help to reconcile contradictory effects reported for AP-2α, in particular in breast tumour studies, and also may explain the partially overlapping yet distinct roles observed for AP-2α, β and γ during development.

## Abbreviations

DCIS: ductal carcinoma *in situ*; EMSA: electromobility shift assay; EST: expressed sequence tag; IAA: iodoacetamide; NEM: N-ethylmaleimide.

## Competing interests

The authors declare that they have no competing interests.

## Authors' contributions

CB carried out the majority of the experiments and drafted the manuscript. KVC set up the cyclin D3 reporter assay. AMP set up the 3XAP-2 Bluc assay. MC contributed to the isoform cloning and to the antibody purifications. AGS performed the EMSA analysis. HCH conceived the study, and participated in its design and coordination and helped to draft the manuscript. All authors read and approved the final manuscript.

## Supplementary Material

Additional file 1 **Supplementary Figures S1 to S5**. A PDF file with each of the supplementary figures referred to in the text, together with an explanatory legend. Click here for file

## References

[B1] Eckert D Buhl S Weber S Jäger R Schorle H The AP-2 family of transcription factors Genome Biol 2005 6 246 10.1186/gb-2005-6-13-246 16420676 PMC1414101

[B2] Friedrichs N Jäger R Paggen E Rudlowski C Merkelbach-Bruse S Schorle H Buettner R Distinct spatial expression patterns of AP-2alpha and AP-2gamma in non-neoplastic human breast and breast cancer Mod Pathol 2005 18 431 438 10.1038/modpathol.3800292 15467710

[B3] Friedrichs N Steiner S Buettner R Knoepfle G Immunohistochemical expression patterns of AP2alpha and AP2gamma in the developing fetal human breast Histopathology 2007 51 814 823 10.1111/j.1365-2559.2007.02887.x 18042070

[B4] Gee JM Robertson JF Ellis IO Nicholson RI Hurst HC Immunohistochemical analysis reveals a tumour suppressor-like role for the transcription factor AP-2 in invasive breast cancer J Pathol 1999 189 514 520 10.1002/(SICI)1096-9896(199912)189:4<514::AID-PATH463>3.0.CO;2-9 10629551

[B5] Pellikainen J Naukkarinen A Ropponen K Rummukainen J Kataja V Kellokoski J Eskelinen M Kosma VM Expression of HER2 and its association with AP-2 in breast cancer Eur J Cancer 2004 40 1485 1495 10.1016/j.ejca.2004.02.020 15196531

[B6] Bosher JM Williams T Hurst HC The developmentally regulated transcription factor AP-2 is involved in c-erbB-2 overexpression in human mammary carcinoma Proc Natl Acad Sci USA 1995 92 744 747 10.1073/pnas.92.3.744 7846046 PMC42696

[B7] Turner BC Zhang J Gumbs AA Maher MG Kaplan L Carter D Glazer PM Hurst HC Haffty BG Williams T Expression of AP-2 transcription factors in human breast cancer correlates with the regulation of multiple growth factor signalling pathways Cancer Res 1998 58 5466 5472 9850080

[B8] Pellikainen JM Kosma VM Activator protein-2 in carcinogenesis with a special reference to breast cancer--a mini review Int J Cancer 2007 120 2061 2067 10.1002/ijc.22648 17330235

[B9] Bragança J Eloranta JJ Bamforth SD Ibbitt JC Hurst HC Bhattacharya S Physical and functional interactions among AP-2 transcription factors, p300/CREB-binding protein, and CITED2 J Biol Chem 2003 278 16021 16029 12586840 10.1074/jbc.M208144200

[B10] Bragança J Swingler T Marques FIR Jones T Eloranta JJ Hurst HC Shioda T Bhattacharya S Human CREB-binding protein/p300-interacting transactivator with ED-rich tail (CITED) 4, a new member of the CITED family, functions as a co-activator for transcription factor AP-2 J Biol Chem 2002 277 8559 8565 11744733 10.1074/jbc.M110850200

[B11] Bamforth SD Braganca J Eloranta JJ Murdoch JN Marques FI Kranc KR Farza H Henderson DJ Hurst HC Bhattacharya S Cardiac malformations, adrenal agenesis, neural crest defects and exencephaly in mice lacking Cited2, a new Tfap2 co-activator Nat Genet 2001 29 469 474 10.1038/ng768 11694877

[B12] Jiang MS Tang QQ McLenithan J Geiman D Shillinglaw W Henzel WJ Lane MD Derepression of the C/EBPalpha gene during adipogenesis: identification of AP-2alpha as a repressor Proc Natl Acad Sci USA 1998 95 3467 3471 10.1073/pnas.95.7.3467 9520389 PMC19859

[B13] Wajapeyee N Britto R Ravishankar HM Somasundaram K Apoptosis induction by activator protein 2alpha involves transcriptional repression of Bcl-2 J Biol Chem 2006 281 16207 16219 10.1074/jbc.M600539200 16533807

[B14] Wang X AP-2: a regulator of EGF receptor signaling and proliferation in skin epidermis J Cell Biol 2006 172 409 421 10.1083/jcb.200510002 16449191 PMC2063650

[B15] Eloranta JJ Hurst HC Transcription factor AP-2 interacts with the SUMO-conjugating enzyme UBC9 and is sumolated *in vivo* J Biol Chem 2002 277 30798 30804 10.1074/jbc.M202780200 12072434

[B16] Meier P Koedood M Philipp J Fontana A Mitchell PJ Alternative mRNAs encode multiple isoforms of transcription factor AP-2 during murine embryogenesis Dev Biol 1995 169 1 14 10.1006/dbio.1995.1121 7750631

[B17] Limesand SW Anthony RV Novel activator protein-2alpha splice-variants function as transactivators of the ovine placental lactogen gene Eur J Biochem 2001 268 2390 2401 10.1046/j.1432-1327.2001.02124.x 11298758

[B18] Davuluri RV Suzuki Y Sugano S Plass C Huang TH The functional consequences of alternative promoter use in mammalian genomes Trends Genet 2008 24 167 177 10.1016/j.tig.2008.01.008 18329129

[B19] Williams T Admon A Lüscher B Tjian R Cloning and expression of AP-2, a cell-type-specific transcription factor that activates inducible enhancer elements Genes Dev 1988 2 1557 1569 10.1101/gad.2.12a.1557 3063603

[B20] Boyes J Byfield P Nakatani Y Ogryzko V Regulation of activity of the transcription factor GATA-1 by acetylation Nature 1998 396 594 598 10.1038/25166 9859997

[B21] Soutoglou E Katrakili N Talianidis I Acetylation regulates transcription factor activity at multiple levels Mol Cell 2000 5 745 751 10.1016/S1097-2765(00)80253-1 10882110

[B22] Hollywood DP Hurst HC A novel transcription factor, OB2-1, is required for overexpression of the proto-oncogene c-erbB-2 in mammary tumour lines EMBO J 1993 12 2369 2375 8099545 10.1002/j.1460-2075.1993.tb05891.x PMC413467

[B23] Debnath J Muthuswamy SK Brugge JS Morphogenesis and oncogenesis of MCF-10A mammary epithelial acini grown in three-dimensional basement membrane cultures Methods 2003 30 256 268 10.1016/S1046-2023(03)00032-X 12798140

[B24] ImageJ http://rsb.info.nih.gov/ij/

[B25] Iacono M Mignone F Pesole G uAUG and uORFs in human and rodent 5'untranslated mRNAs Gene 2005 349 97 105 10.1016/j.gene.2004.11.041 15777708

[B26] Gong L Kamitani T Fujise K Caskey LS Yeh ET Preferential interaction of sentrin with a ubiquitin-conjugating enzyme, Ubc9 J Biol Chem 1997 272 28198 28201 10.1074/jbc.272.45.28198 9353268

[B27] Vernimmen D Begon D Salvador C Gofflot S Grooteclaes M Winkler R Identification of HTF (HER2 transcription factor) as an AP-2 (activator protein-2) transcription factor and contribution of the HTF binding site to ERBB2 gene overexpression Biochem J 2003 370 323 329 10.1042/BJ20021238 12418962 PMC1223148

[B28] Arpino G Wiechmann L Osborne CK Schiff R Crosstalk between the estrogen receptor and the HER tyrosine kinase receptor family: molecular mechanism and clinical implications for endocrine therapy resistance Endocr Rev 2008 29 217 233 10.1210/er.2006-0045 18216219 PMC2528847

[B29] Geiss-Friedlander R Melchior F Concepts in sumoylation: a decade on Nat Rev Mol Cell Biol 2007 8 947 956 10.1038/nrm2293 18000527

[B30] Ramos YF Hestand MS Verlaan M Krabbendam E Ariyurek Y van Galen M van Dam H van Ommen GJ den Dunnen JT Zantema A 't Hoen PA Genome-wide assessment of differential roles for p300 and CBP in transcription regulation Nucleic Acids Res 38 5396 5408 10.1093/nar/gkq184 20435671 PMC2938195

