# Supplementary material for: Alternative TFAP2A isoforms have distinct activities in breast cancer
Source: Breast Cancer Res. 2011 Mar 4;13(2):R23. doi: 10.1186/bcr2838 (PMC3219183; doi:10.1186/bcr2838)
Supplement: Additional file 1 — Supplementary Figures S1 to S5. A PDF file with each of the supplementary figures referred to in the text, together with an explanatory legend. [file bcr2838-S1.PDF]

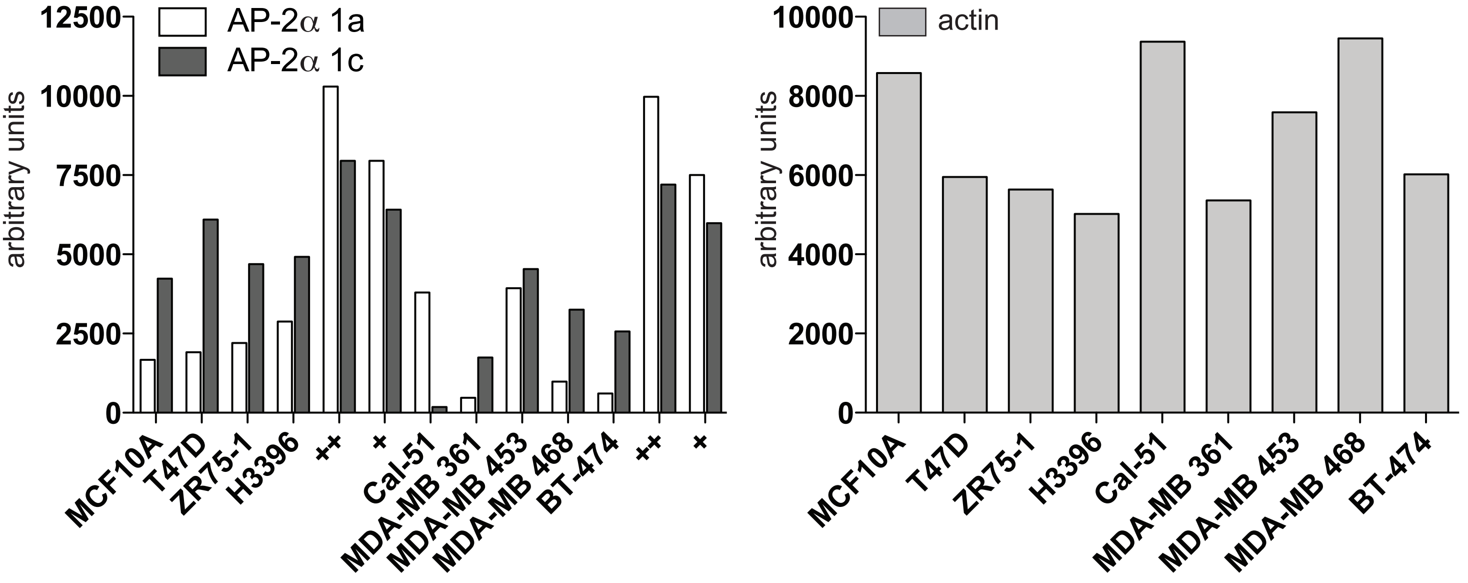

Supplementary Figure S1: Quantification of AP-2α isoform 1a and 1c protein expression level.
The intensity of signal relative to the Western blots shown in Figure 2c was quantified with ImageJ and is graphically represented for AP-2α 1a, 1c and actin, as indicated.

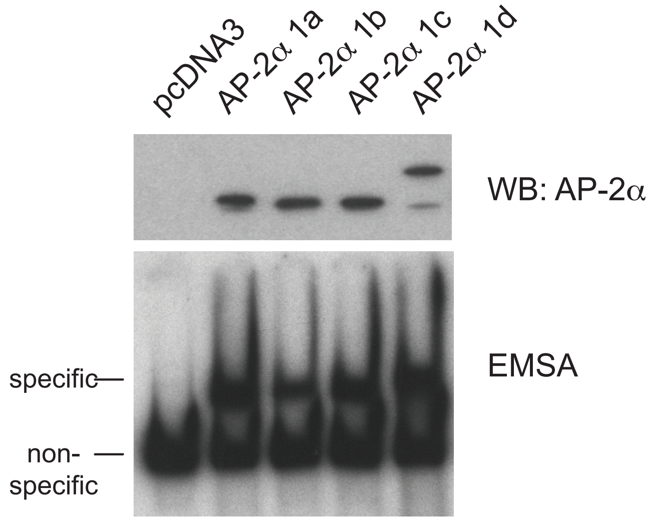

Supplementary Figure S2: AP-2α isoforms have similar binding affinity for an AP-2α consensus binding site.
HepG2 cells were transfected with the expression vector pcDNA3 encoding the different isoforms. 5 µg of nuclear extracts were analysed by Western blot with a AP-2α antibody, or analysed by EMSA with the oligonucleotide containing a well-characterised consensus AP-2 binding site from the human MTIIA promoter (Scibetta et al., 2010).

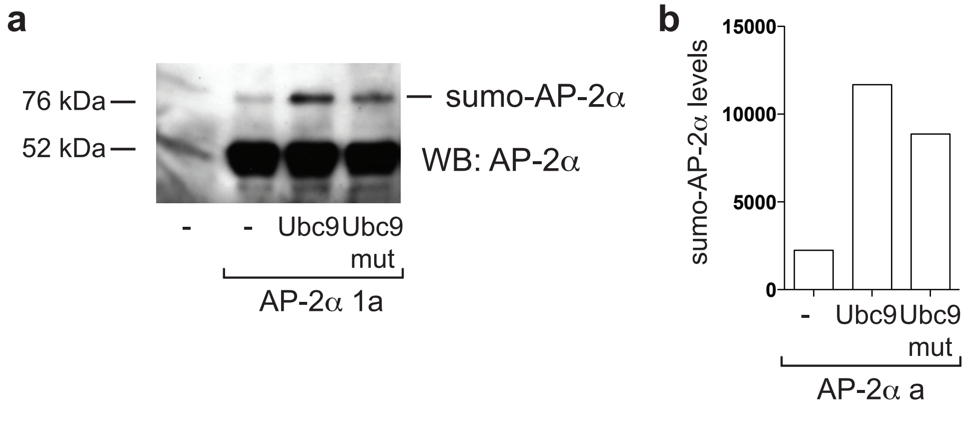

Supplementary Figure S3: Ubc9 C93S overexpression diminishes sumoylation of AP-2α isoform 1a.
(**a**), (**b**): HepG2 cells were transfected with 0.3 µg/well pcDNA3-AP-2α isoform 1a, 0.6 µg/well pSG-Ubc9 and 0.3 µg/well pSG-SUMO1, thus maintaining the same ratio used in the experiment illustrated in figure 6c. Lysates were collected 48h after transfection in RIPA buffer containing IAA and NEM. Lysates were analysed by western blot with an anti-AP-2α (3B5) antibody (**a**). The intensity of the sumoylated band as determned with ImageJ is represented in (**b**) (arbitrary units).

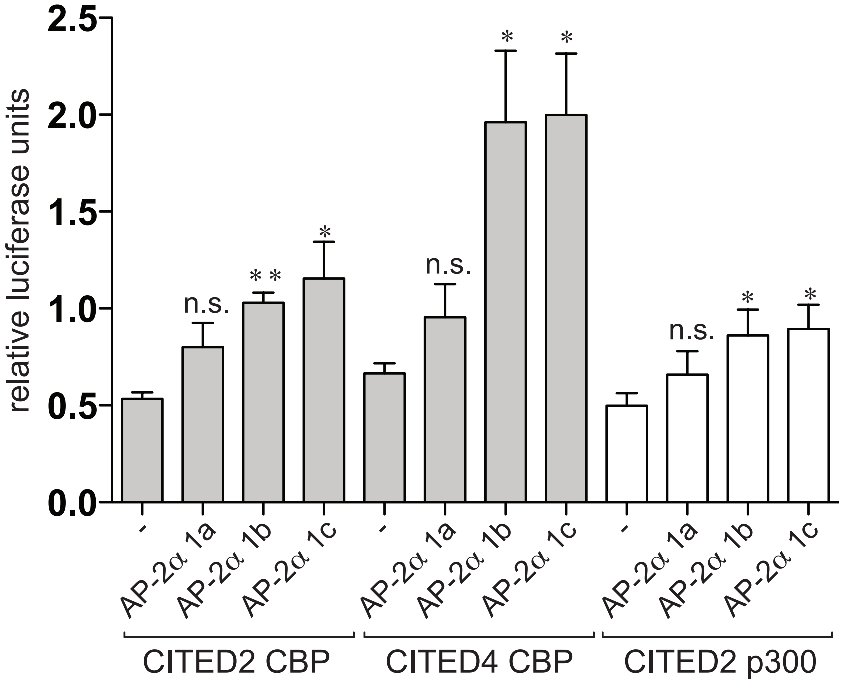

Supplementary Figure S4: AP-2α 1a is the weakest activator of the ERBB2 promoter. HepG2 cells were transfected with 0.12 µg/well p500 ErbB2-luc, 0.02 µg/well renilla hRG, 0.06 µg/well pCI-p300 or pRC/CMV CBP, 0.06 µg/well pcDNA3-CITED2 or CITED4, and 0.06 µg/well of the different pcDNA3-AP-2α constructs. The average and standard error of three independent experiments is represented. Cotransfection with CITED4 and p300 did not result in significant transactivation with any of the isoforms(not shown). Results shown in Figure 6 for CITED2/p300 at the corresponding ratio (1:0.5) are shown for comparison (open bars).

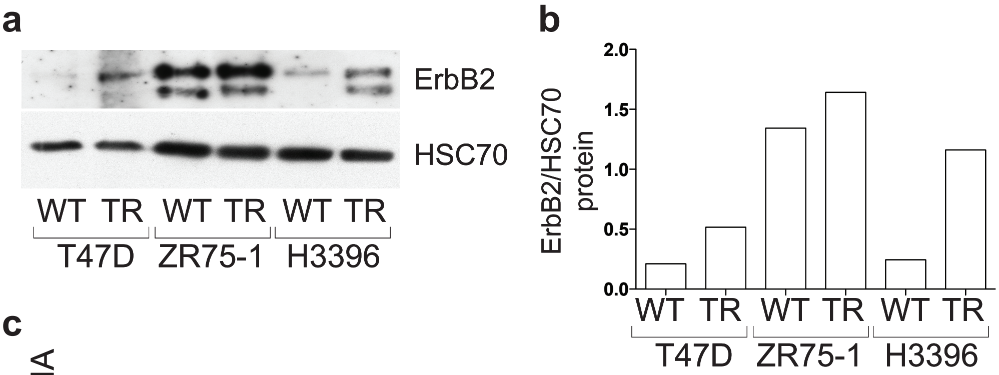

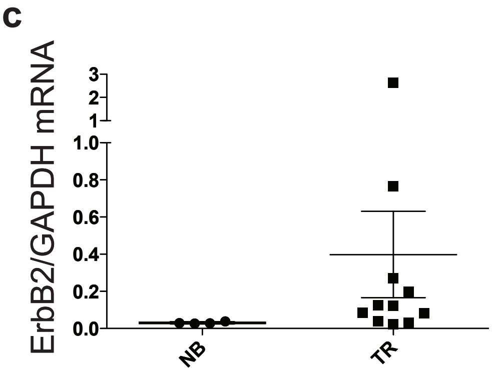

Supplementary Figure S5: ErbB2 levels are elevated in tamoxifen resistant cell lines and tumour samples.
(**a**), (**b**): Levels of ErbB2 were determined by Western Blot in different wild-type cell lines and in their tamoxifen-resistant counterparts. The amount of signal, normalised by HSC70 signal, is graphically represented in (**b**).
(**c**): Normal breast (4) and tumour samples (10) were analysed by real-time PCR for ErbB2 expression levels and normalised by GAPDH expression. The difference is statistically significant (p=0.043, Mann-Whitney test).
